# Synthesis of Bioactive Chlorogenic Acid-Silica Hybrid Materials via the Sol–Gel Route and Evaluation of Their Biocompatibility

**DOI:** 10.3390/ma10070840

**Published:** 2017-07-21

**Authors:** Michelina Catauro, Severina Pacifico

**Affiliations:** 1 Department of Industrial and Information Engineering, University of Campania “Luigi Vanvitelli”, Via Roma 29, 81031 Aversa, Italy; 2 Department Environmental, Biological and Pharmaceutical Sciences and Technologies, University of Campania “Luigi Vanvitelli”, Via Vivaldi 43, 81100 Caserta, Italy; severina.pacifico@unicampania.it

**Keywords:** sol–gel method, organic-inorganic hybrids, chlorogenic acid, cytotoxicity, biocompatibility

## Abstract

Natural phenol compounds are gaining a great deal of attention because of their potential use as prophylactic and therapeutic agents in many diseases, as well as in applied science for their preventing role in oxidation deterioration. With the aim to synthetize new phenol-based materials, the sol–gel method was used to embed different content of the phenolic antioxidant chlorogenic acid (CGA) within silica matrices to obtain organic-inorganic hybrid materials. Fourier transform infrared (FTIR) measurements were used to characterize the prepared materials. The new materials were screened for their bioactivity and antioxidant potential. To this latter purpose, direct DPPH (2,2-diphenyl-1-picrylhydrazyl) and ABTS (2,2′-azinobis-(3-ethylbenzothiazolin-6-sulfonic acid) methods were applied: radical scavenging capability appeared strongly dependent on the phenol amount in investigated hybrids, and became pronounced, mainly toward the ABTS radical cation, when materials with CGA content equal to 15 wt% and 20 wt% were analyzed. The in vitro biocompatibility of the synthetized materials was estimated by using the MTT assay towards fibroblast NIH 3T3 cells, human keratinocyte HaCaT cells, and the neuroblastoma SH-SY5Y cell line. As cell viability and morphology of tested cell lines seemed to be unaffected by new materials, the attenuated total reflectance (ATR)-FTIR method was applied to deeply measure the effects of the hybrids in the three different cell lines.

## 1. Introduction

The growing interest in plants’ secondary metabolites is due to their ability to be bioactive compounds with pharmacological or toxicological effects in humans and animals. Indeed, these naturally-occurring substances, also known as phytochemicals, are still the main source of lead molecules in modern drug discovery and development, and their evidenced health promoting benefits in counteracting chronic and degenerative pathologies (e.g., cancer, cardiovascular, and neurodegenerative diseases), make the natural products research an endless and intriguing research field with multidisciplinary approach [1]. Among phytochemicals, phenol, and polyphenols have gained a great deal of importance due to their antioxidant capability, which allows them to exert preventive and protectant effects in human cells [2,3]. Indeed, the highly-acclaimed chemopreventive role of these substances appear to be related to their ability to induce dose-dependent oxidative stress, DNA damage, and apoptosis in tumor, but not normal tissue [4,5].

**Figure 1:**
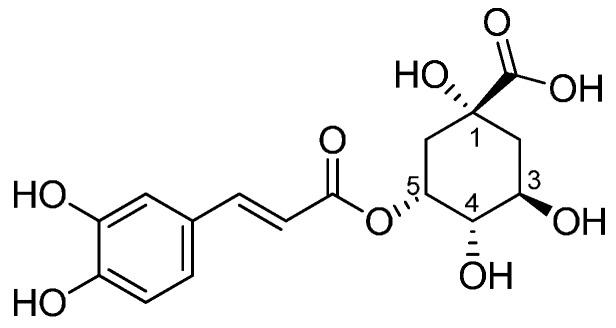
Chemical structure of chlorogenic acid (CGA).

5-O-caffeoylquinic acid, better known as chlorogenic acid (CGA), is a hydroxycinnamoyl derivative, whose structure consists of a caffeic acid moiety esterified with (−)-quinic acid (Figure 1).

This dietary metabolite, broadly distributed in edible plants, possesses many health-promoting properties [6]. Accumulating evidence demonstrated that CGA possesses antibacterial, anti-inflammatory, and anti-oxidant activities [7], as well as appearing to be an effective chemopreventive agent. The growing interest in the dietary supplementation of CGA as a nutraceutical agent in food formulations, due to its various medicinal properties, its low bioavailability and stability, addressed the encapsulation of CGA in a variety of polymers and the synthesis of CGA loaded chitosan nanoparticles with preserved antioxidant activity [8]. Biomaterial science was further fascinated by this molecule and recently CGA-gelatin was prepared as a coating for the preservation of seafood [9]. Moreover, highly-adhesive bioinspired polyurethanes based on CGA were prepared from 4,4′-methylenebis (cyclohexyl isocyanate) and polyethylene glycol 200 providing biocompatible-adhesive bioinspired polyurethanes, which appeared to be good candidates for medical applications as a tissue adhesive material. The sol–gel technique was employed for the preparation of carbon composite electrode modified with electroless deposition of chlorogenic acid, which were evaluated for their stability and electrochemical properties was [10]. The high versatility of sol–gel routes for the formulation of organic-inorganic hybrid materials [11,12,13], together with data from our recent researches aimed at entrapping another natural antioxidant compound, such as quercetin, in a silica matrix [14,15,16], intrigued us to investigate the possibility of synthesizing new materials having chlorogenic acid as the organic component. Thus, silica-based materials, differing in their CGA amount (5 wt%, 10 wt%, 15 wt%, and 20 wt%), were synthetized and characterized by Fourier transform infrared spectroscopy (FTIR) and UV-VIS spectroscopy. The preservation of CGA antioxidant chemical features was demonstrated by applying DPPH and ABTS tests. Bioactivity was studied by soaking the samples into a simulated body fluid (SBF) and evaluating the formation of a hydroxyapatite layer on their surface by FTIR spectroscopy and scanning electron microscopy (SEM) after 21 days of exposure. Biocompatibility was assessed by the MTT direct contact test using murine fibroblast NIH 3T3 cell line, human keratinocyte HaCaT cells and neuroblastoma SH-SY5Y cell line. The choice of cell lines was deliberate. Fibroblasts are cell types that interact with proteins on biomaterials surfaces, playing important roles in biomaterials rejection and implant failure. The HaCaT cell line, although obviously immortal, is a non-tumorigenic cell line [17]. On the other hand, considering the ability of CGA to act as an antioxidant at low doses and pro-oxidant at high doses, SH-SY5Y cells, particularly sensitive to oxidative stress onset, were also used. In fact, the brain is highly vulnerable to oxidative stress due to its high O_2_ consumption, its modest antioxidant defenses and its lipid-rich constitution. Attenuated total reflectance (ATR)-FTIR analyses were also applied to deeply measure the effects of the hybrids in the treated cell lines.

## 2. Results and Discussion

Recently, in the search for new biocompatible biomaterials able to provide antioxidant functionality and to not exacerbate the body’s normal oxidant and inflammatory response, our research group has optimized the synthesis of novel intrinsically-antioxidant quercetin-based biomaterials, which could be employed in dentistry, as components of glass ionomer cement, and in medicine, as replacements for bone implants. With the aim at preparing new bioactive and biocompatible organic-inorganic hybrids, our attention is turned to chlorogenic acid, a small phenol compound, broadly investigated for its several bioactive and health-promoting properties, which appears as an interesting compound to be incorporated into pharmaceutical, cosmetic, or food products.

### 2.1. Characterization of Synthetized Organic-Inorganic Hybrid Materials

**Figure 2:**
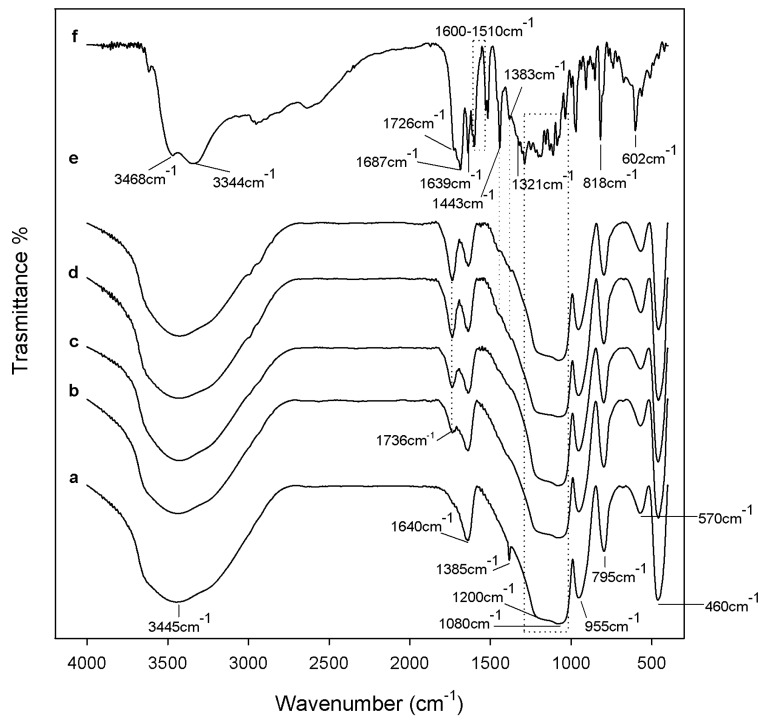
FT-IR spectra of (**a**) pure SiO_2_; (**b**) SiO_2_/CGA, 5 wt%; (**c**) SiO_2_/CGA, 10 wt%; (**d**) SiO_2_/CGA, 15 wt%; (**e**) SiO_2_/CGA, 20 wt%; and (**f**) pure CGA.

Figure 2 shows the spectra of the SiO_2_/CGA hybrids (curve from b to e) compared to the spectra of the pure SiO_2_ (curve a) and CGA (curve f). The spectrum of the pure SiO_2_ (curve a) shows all the typical peaks of the silica sol–gel materials [18,19,20].

**Figure 3:**
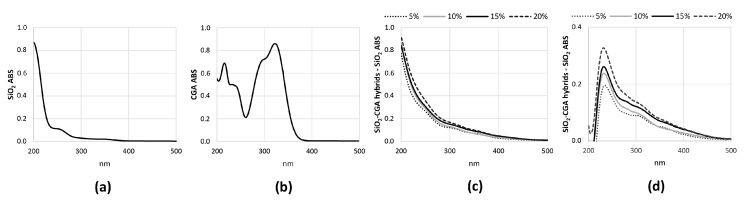
UV-VIS spectra of (**a**) pure SiO_2_; (**b**) pure CGA; (**c**) SiO_2_/CGA hybrids. Panel (**d**) shows different UV-VIS spectra obtained by importing the acquired data into Excel and subtracting SiO_2_ signals from the SiO_2_ scan to those of each hybrid.

The broad intense band at 3445 cm^−1^ and the peak at 1640 cm^−1^ are due to –OH stretching and bending vibrations in the hydration water. The bands at 1080 cm^−1^ and 795 cm^−1^ and the shoulder at 1200 cm^−1^ are associated to the asymmetric and symmetric Si–O stretching vibrations. The signal at 460 cm^−1^ is due to the bending of the Si–O–Si bonds. Moreover, three peaks generally observed in alkoxy-derived silica gels are visible at 1385 cm^−1^, 955 cm^−1^ and at 570 cm^−1^, which are due to residual nitrate anions [21], Si–OH bonds, and four-fold siloxane residual cyclic structures in the silica network, respectively [18,19,22]. The chlorogenic acid IR spectrum shows OH groups stretching at 3468 and 3344 cm^−1^, whereas OH bending of the phenol function was at 1383 cm^−1^. The band assigned to the stretching C=O vibration of the carboxylic group is located at 1726 cm^−1^, whereas the band at 1687 cm^−1^ is due to the stretching C=O vibrations of the ester group [23]. The stretching vibration of the C=C fragment is at 1639 cm^−1^, whereas the bands derived mainly from stretching vibrations of the aromatic ring are in the range of 1600–1510. The band at 1443 cm^−1^, which can be assigned to a phenyl ring stretch, was previously attributed to the C_3_–O–H group, whose contribution was found equal to 64% [24]. The in-plane bending band of C–H in the aromatic hydrocarbon is detectable at 1321 cm^−1^, and out-of-plane bending bands are at 818 and 602 cm^−1^. The spectra of the SiO_2_/CGA hybrids show all the described SiO_2_ peaks, whereas it lacks nitrate signals. Indeed, a broadening of the SiO_2_ strong band at 1080 cm^−1^ occurs, together with a marked increase in the intensity of the shoulder at 1200 cm^−1^, as a result of the contribution of the several, intense signals of the phenyl ring and C–O–C bonds in CGA (see curve f), which are present in this spectral region. The observation of two weak bands at 1447 and 1373 cm^−1^ in the spectrum of all the hybrid samples, and are clearer in those containing 15 wt% and 20 wt% of CGA (curves d, e), corresponding to 1443 cm^−1^ and 1383 cm^−1^ in the CGA spectrum, which seemed to confirm this hypothesis. In fact, these signals, whose wavenumber are displaced by 4 cm^−1^ and 10 cm^−1^ with respect to those detected in CGA, are ascribable to phenyl ring stretching and phenol bending vibrations [9]. The displacement of the CGA carboxylic group C=O stretch by about 10 cm^−1^ (1736 cm^−1^) and the observation of a weak shoulder at a lower wavenumber, attributable to the ester C=O stretch vibration, allowed us to hypothesize the establishment of H-bonds with the SiO_2_ inorganic matrix. FTIR data suggested the synthesis of materials in which chlorogenic acid was embedded in the silica network. UV-VIS spectra, recorded on extracts obtained by swelling powders of investigated materials in water, strengthen this hypothesis (Figure 3). In SiO_2_ spectrum, two absorption bands were also observed, the first one located at 204 nm corresponds to electronic transitions exhibited by sol–gel SiO_2_ materials [25], while the weak other one (260 nm) could be the result of the applied acid-catalyzed sol–gel route (Figure 3a). For CGA, maximum absorbances occurred at 217 nm (with shoulder at 240 nm) and at 324 nm (with shoulder at 296 nm), whereas the minimum point was at 262 nm (Figure 3b). The UV spectra of hybrids were in accordance with silica-induced changes of the chlorogenic acid skeleton, which modified the characteristic different electronic transitions of the caffeoyl quinic acid (Figure 3c,d).

### 2.2. Bioactivity Test

**Figure 4:**
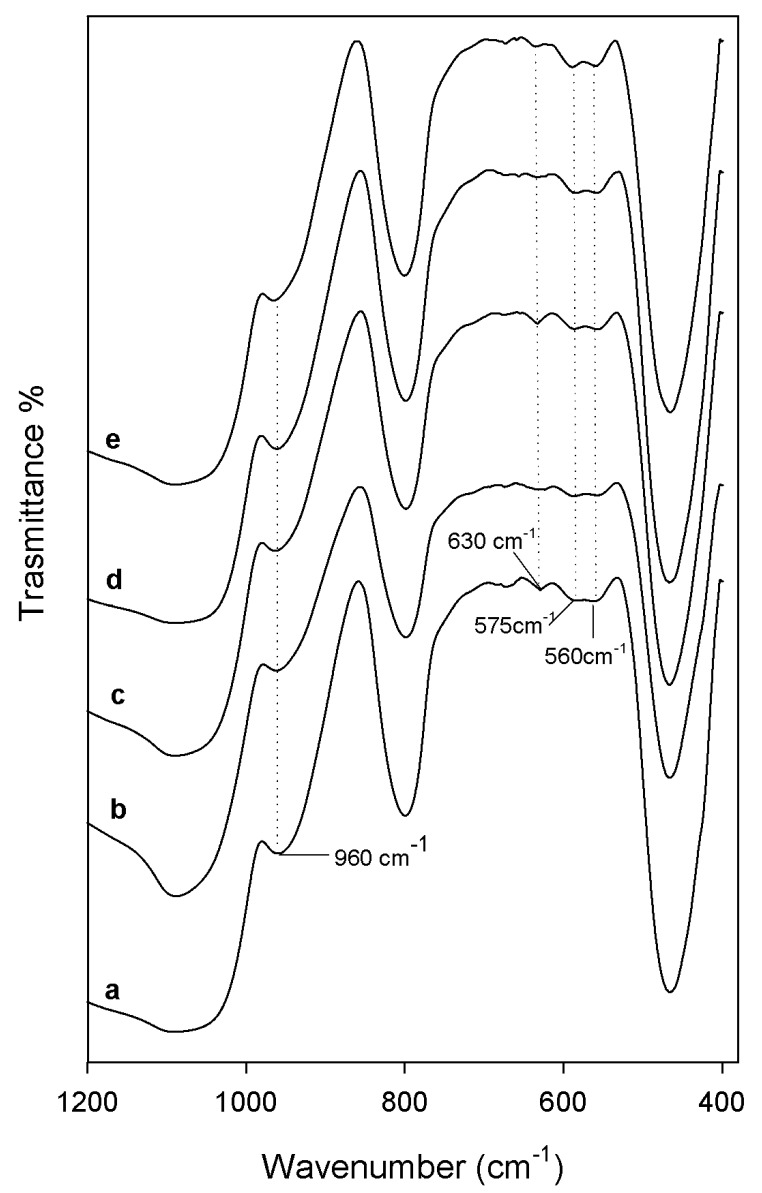
FT-IR spectra of (**a**) pure SiO_2_; (**b**) SiO_2_/CGA, 5 wt%; (**c**) SiO_2_/CGA, 10 wt%; (**d**) SiO_2_/CGA, 15 wt%; and (**e**) SiO_2_/CGA, 20 wt% after 21 days of exposure to SBF.

After 21 days of soaking into the SBF solution, both the sample powders and sample disks were air dried and their ability to induce the nucleation of a hydroxyapatite layer on their surface was evaluated by FTIR (Shimadzu, Tokyo, Japan) and SEM (Quanta 200, FEI, Eindhoven, The Netherlands) analyses, respectively. Comparing FTIR spectra of the sample powders before (Figure 2) and after the exposure to SBF (Figure 4), a new peak at 630 cm^−1^ and the split of the band at 570 cm^−1^ in two new peaks at 575 cm^−1^ and 560 cm^−1^ were observed. Those spectra modifications could be ascribable to the formation of the hydroxyapatite precipitate and, in particular, to the stretching of the hydroxyapatyte –OH groups and the vibrations of PO_4_^3−^ groups, respectively [26,27]. Moreover, a slight up-shift of the Si–OH band (from 955 cm^−1^ to 960 cm^−1^) suggest the interaction of the hydroxyapatite layer with the –OH groups of the silica matrix.

**Figure 5:**
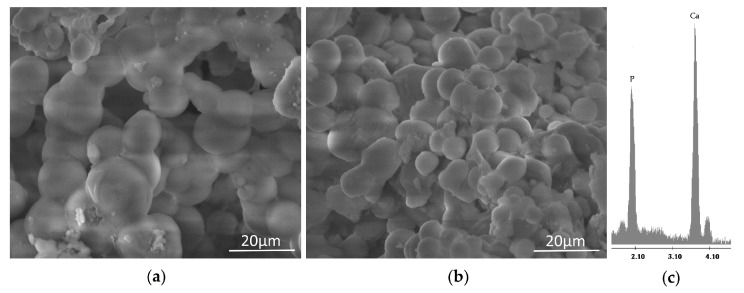
SEM micrographs of (**a**) pure SiO_2_ and (**b**) a representative SiO_2_/CGA hybrid; and (**c**) EDX analysis.

The formation of the hydroxyapatite precipitate on the sample surfaces was confirmed by SEM imagery (Figure 5). After 21 days of exposure to SBF, indeed, the surfaces of all samples appear covered by a precipitate with the globular shape typical of hydroxyapatite [28]. No difference was detected in the distribution and amount of precipitate, as the whole surface of the samples is covered by the globules. Therefore, only representative SEM micrographs of the SiO_2_ and SiO_2_/CGA systems are reported (Figure 5a,b). The energy-dispersive X-ray (EDX) (Quanta 200, FEI, Eindhoven, The Netherlands) microanalysis (Figure 5c) confirmed that the globules consist of Ca and P in an atomic ratio equal to 1.67.

### 2.3. Antiradical Capability of SiO_2_-CGA Hybrids

**Figure 6:**
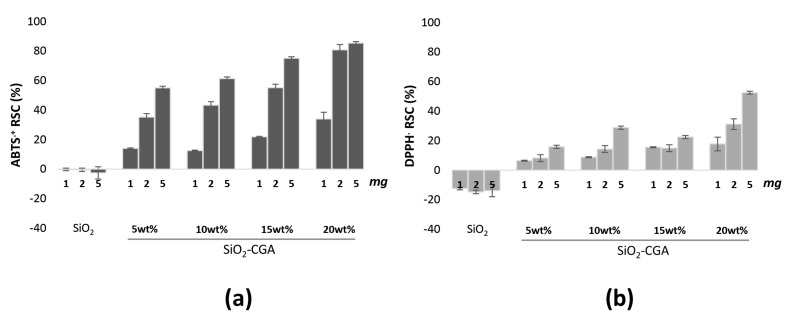
Radical Scavenging Capacity (RSC, %) of different amounts of SiO_2_-CGA hybrids, and SiO_2_ samples towards (**a**) ABTS^•+^ and (**b**) DPPH^•^. Values, reported as percentage vs. a blank, are the mean ± SD of measurements carried out on three samples (*n* = 3) analyzed three times.

DPPH^•^ and ABTS^•+^ methods, which use two radical probes which may be neutralized by the transfer of an electron and/or a hydrogen atom, allowed us to evaluate the radical scavenging capacity of the synthetized hybrids and to compare it to that exercised by pure chlorogenic acid. Caffeoyl quinic acid silica-based materials were able to exert an anti-radical power strongly dependent on the phenol concentration therein, reaching its maximum effect when the highest chlorogenic acid dose level (20 wt%) was embedded (Figure 6). Analogously, the amount of hybrids placed in contact with the probe solutions seemed to affect the antiradical response, which appeared far below that elicited by pure chlorogenic acid. This latter, which is known to display notable free radical scavenging effects, showed ID_50_ values of 0.53 and 6.06 μg/mL vs. DPPH^•^ and ABTS^•+^, respectively [29]. The scavenging efficiency of pure CGA, as well as of other phenol compounds exhibiting similar structural features, is commonly ascribed to the two exchangeable hydrogen atoms (those of catechol moiety), whose presence makes phenol compounds biologically-reactive molecules capable of exhibiting both anti- and pro-oxidant behavior.

### 2.4. Cytotoxicity of SiO_2_-CGA Hybrids

**Figure 7:**
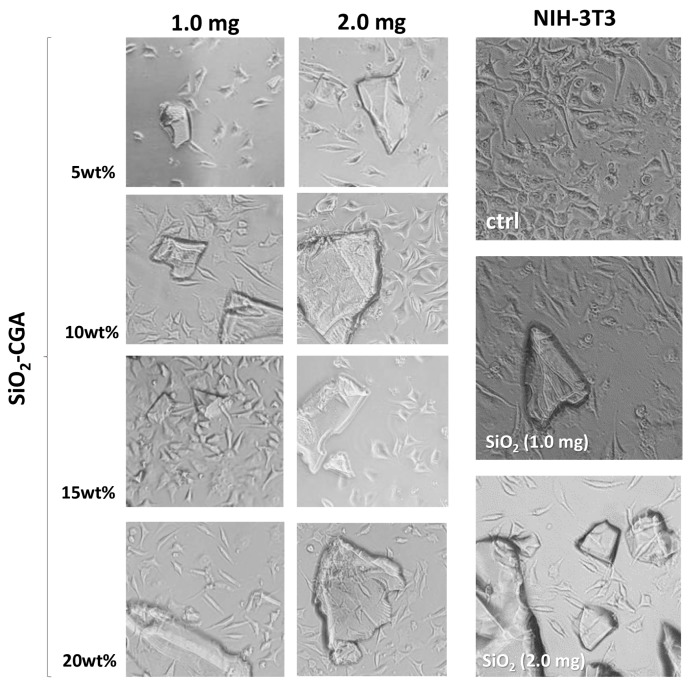
Morphological changes in hybrids- and SiO_2_-treated NIH-3T3 cells. Representative images were acquired by an inverted phase contrast brightfield Zeiss Primo Vert Microscope. Ctrl = untreated cells.

In order to assess the influence of the synthesized hybrid materials on morphology and cell proliferation, NIH-3T3 murine fibroblast, HaCaT human keratinocyte, and SH-SY5Y human neuroblastoma cell lines were grown in the presence of powders of the investigated materials. After 48 h exposure, the MTT cytotoxicity assay was performed. In Figure 7 morphological changes detected directly from NIH-3T3 culture plates with a phase-contrast microscope are reported. The synthetized materials did not seem to affect NIH-3T3 cell morphology.

**Figure 8:**
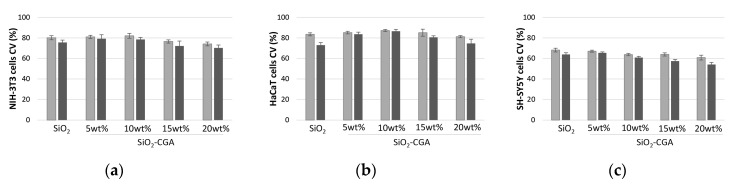
Cell Viability (CV, %) of (**a**) NIH-3T3, (**b**) HaCaT, and (**c**) SH-SY5Y cells treated with ■ 1.0 mg, and ■ 2.0 mg of SiO_2_-CGA hybrids, after 48 h exposure time by means of MTT test results. Values, reported as percentage vs. an untreated control, are the mean ± SD of measurements carried out on three samples (*n* = 3) analyzed six times.

The proliferation of the embryonic fibroblast cells was observed to increase depending on the content of the embedded phenol (Figure 8a). In particular, it reached its maximum percentage value when SiO_2_-CGA, 10 wt% was tested, whereas a weak decrease in cell viability was observed in cells treated with SiO_2_-CGA, 15 wt%, and SiO_2_-CGA, 20 wt% samples.

Similar behavior was observed for HaCaT cells, which strongly preserved the morphology after the treatment with the investigated hybrids. As for NIH-3T3 cells, MTT data were in accordance with a mild in vitro suppression of HaCaT cells’ mitochondrial redox activity to levels that would be acceptable based on standards used to evaluate alloys and composites (<25% suppression of dehydrogenases activity; Figure 8b) [30]. Thus, SiO_2_/CGA hybrids were found biocompatible towards non-tumorigenic NIH-3T3 and HaCaT cell lines, highlighting that the adopted synthesis strategy provided materials in which the establishment of a network between the phenol compound and the silica matrix was conducive to maintaining antioxidant functionality of the organic component, inhibiting the dose-dependent anti-proliferative efficacy, commonly observed when high doses of chlorogenic acid were tested.

Recently-published findings from pre-clinical experimental and phase I clinical studies have shown that treatment with CGA has shown therapeutic effects in breast cancer, brain tumors, lung cancer, colon cancer, and chronic myelogenous leukemia [31]. The ability of chlorogenic acid to exert an anti-tumor effect in multiple malignant tumors appeared to be shared by synthetized hybrids towards neuroblastoma SH-SY5Y cells, which seemed to change their phenotype, exhibiting a decrease in proliferation dependent on both the phenol amount embedded and the dose of hybrid directly placed in contact with them (Figure 8c).

In fact, when a dose equal to 2.0 mg of SiO_2_-CGA, 20 wt% was tested, mitochondrial redox activity was inhibited by 49.9%. A marked dose-dependent anti-proliferative activity was found for pure chlorogenic acid, which was able to inhibit SH-SY5Y cell viability by 50% at a concentration level equal to 31.1 μg/mL. Thus, the embedment of high doses of chlorogenic acid in silica matrix, while massively preserving the cell growth of treated cells with respect to the pure compound, seemed to provide a material able to exert pro-oxidant activity. This hypothesis was supported by the experimental data of several studies, which highlights that dietary phenols and polyphenols can potentially confer additional benefits, but high-doses may elicit toxicity, thereby establishing a double-edged sword in their use as supplements [32].

**Figure 9:**
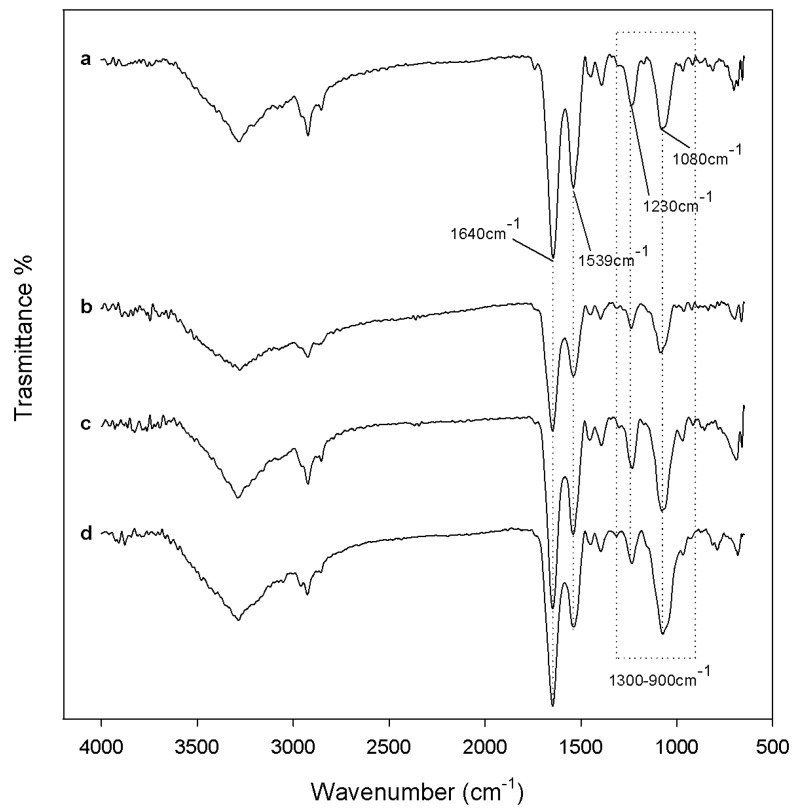
Representative ATR-FTIR spectra of cell suspensions (**a**) untreated or previously treated with (**b**) pure SiO_2_; (**c**) SiO_2_/CGA, 5 wt%; and (**d**) SiO_2_/CGA, 15 wt%.

In order to unravel the mechanism underlying the observed cytotoxicity, ATR-FTIR analyses were carried out [33]. The spectra, acquired in the 650–4000 cm^−1^ region of cell suspensions untreated or previously treated with synthetized hybrids dose are depicted in Figure 9. ATR-FTIR spectra of viable, apoptotic, and necrotic cells are dominated by bands assigned to protein absorption modes: the amide I band is the most intense, centered near 1640 cm^−1^, which corresponds to the C=O stretching vibration coupled to the N–H bending and to C–N stretching modes of peptide bonds [33]. The amide II band at 1539 cm^−1^ is due to vibrational modes involving the C–N–H bending and C–N stretching of the peptide bonds [34]. The spectral analysis showed some significant differences between viable and apoptotic cells. The spectra of apoptotic cells, compared to vital ones, showed a significant decrease in the region between 900 and 1300 cm^−1^. The complexity in this spectral region was due to the contribution of nucleic acids (DNA, RNA), carbohydrates, and phosphates. The band centered at 1080 cm^−1^ was assigned to the symmetric stretching mode of phosphodiesteric bonds in nucleic acids, whereas the band at 1230 cm^−1^ originated from the asymmetric stretching of the same bonds. The occurrence of apoptosis defined an important decrease of the intensity of the region assigned to nucleic acids, compared to that of the amide bands. In particular, the calculated nucleic acids/amide II area ratio, which was equal to 1.07 for untreated cells, was massively reduced to 0.77, 0.65, and 0.45 in cells directly placed in contact with SiO_2_ powder, SiO_2_-CGA, 5 wt%, and SiO_2_-CGA, 15 wt%, respectively. Obtained data were consistent with those reported by Gasparri and Muzio [33] who assessed that the area between 1000 and 1140 cm^−1^, relative to nucleic acids, was the one with the most prominent differences and was, therefore, indicated as a marker of apoptosis. The second detected difference was the increase of the ratio between the area of amide I and amide II peaks. The increase could be due to a cleavage of cellular proteins by different caspases, to the modulation of chaperone activity and of proteasome function or to cytoplasmic acidification, processes that occur during the whole process of apoptosis.

## 3. Materials and Methods

### 3.1. Sol–gel Synthesis

**Figure 10:**
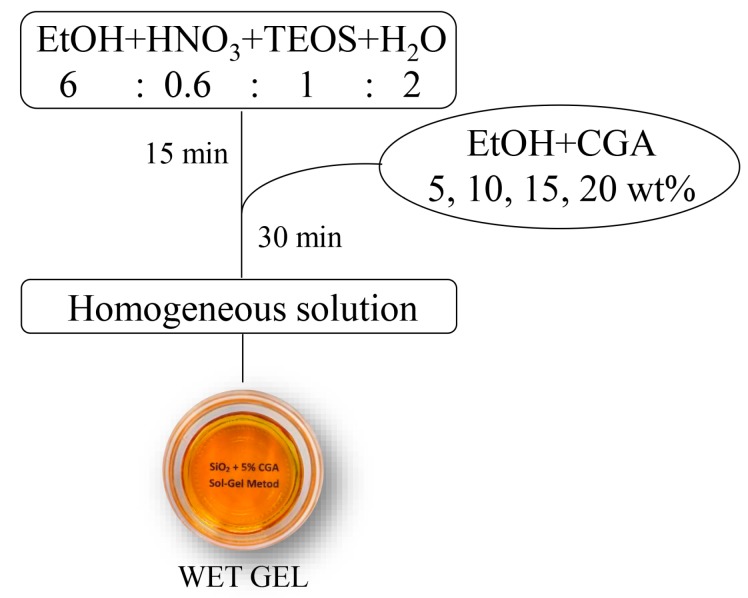
Flowchart of the sol–gel process used to synthesize the SiO_2_-CGA hybrids.

The organic-inorganic hybrids materials, consisting of a SiO_2_ inorganic matrix and different content (5 wt%, 10 wt%, 15 wt%, and 20 wt%) of organic chlorogenic acid (CGA), were synthesized by means of a sol–gel route. A solution of tetraethyl orthosilicate (TEOS, reagent grade, 98%, Sigma Aldrich, Milan, Italy) was used as precursor of the SiO_2_ inorganic matrix. Water was added drop by drop to a solution of TEOS and nitric acid (solution 65%, Sigma Aldrich, Milan, Italy) in ethanol 99% (Sigma Aldrich, Milan, Italy) under stirring. The molar ratio among the reagents in the obtained solution are: EtOH/TEOS = 6, TEOS/HNO_3_ = 1.7, H_2_O/TEOS = 2. After 20 min under stirring, a solution of CGA in pure ethanol was added drop by drop to the prepared TEOS solution under stirring. After 20 min the stirrer was stopped and the prepared solutions were left to gel at room temperature. The gels, then, were put into an oven at 40 °C to allow the removal of the solvent residue avoiding the thermal degradation of the drug. A flowchart of the sol–gel process is reported in Figure 10.

### 3.2. Materials Characterization

The chemical structure of the synthetized materials was investigated by FTIR. A Prestige 21 (Shimadzu, Tokyo, Japan) system, equipped with a DTGS KBr (deuterated tryglycine sulphate with potassium bromide windows) detector allowed us to record the transmittance spectra in the 400–4000 cm^−1^ region, with resolution of 2 cm^−1^ (45 scans). 2 mg of sample powder, mixed with 198 mg of KBr, was then compacted into discs under a pressure of 7 t by using a hydraulic press (Specac, Ltd., Orpington, UK). The FTIR spectra were elaborated by Prestige software (1.30 IRSolution, Shimadzu, Tokyo, Japan).

The UV–VIS spectra of extracts form hybrid materials were also recorded. To this purpose, 1.0 mg of powder of each investigated material underwent ultrasound assisted maceration (Advantage Plus model ES, Darmstadt, Germany) for 1 h using distilled water (2.0 mL) as extracting solvent. Aqueous extracts were then centrifuged at 4500 rpm for 5 min. Supernatants were collected and their spectra acquired in the range 200–500 nm using a UV-1700 spectrophotometer from Shimadzu (Kyoto, Japan).

### 3.3. Bioactivity Test

The bioactivity of the synthesized materials was investigated by evaluating their ability of inducing the hydroxyapatite nucleation when soaked in a simulated body fluid (SBF) solution. SBF is a solution with ion concentrations nearly equal to those in human blood plasma [28]. This was prepared by dissolving NaCl, NaHCO_3_, KCl, MgCl_2_·6H_2_O, CaCl_2_, Na_2_HPO_4_, and Na_2_SO_4_ (Sigma-Aldrich, Milan, Italy) in ultra-pure water buffered at pH 7.4 using 4-(2-hydroxyethyl) piperazine-1-ethanesulfonic acid hemi-sodium salt (HEPES, Sigma-Aldrich).

The dried gels were grinded in a mortar to obtain powders. A part of those powders were pressed by a hydraulic press (Specac, Orpington, UK) to obtain disks with a diameter of 13 mm and a thickness of 2 mm.

The disks and the powders were soaked in SBF within polystyrene bottles, which were placed in a water bath at 37.0 ± 0.5 °C. Taking into account that the ratio of the exposed surface to the volume solution influences the reaction, a constant ratio was maintained as reported in literature [26,28]. The solution was replaced every two days to avoid depletion of the ionic species in the SBF due to the nucleation of biominerals on the samples. After 7, 14, and 21 days of exposure to the SBF solution, the samples were gently rinsed and dried in a glass desiccator. The ability of forming an apatite layer on the surface of both powders and disks after each time of exposure to SBF was investigated by FTIR analysis and using a Quanta 200 SEM (FEI, Eindhoven, The Netherlands), equipped with energy-dispersive X-ray (EDX), respectively.

### 3.4. Determination of DPPH Scavenging Capacity

In order to estimate the DPPH^•^ (2,2-diphenyl-1-picrylhydrazyl) scavenging capability, investigated matrices (1.0, 2.0, and 5.0 mg) were directly added to a DPPH^•^ methanol solution (9.4 × 10^‒5^ M; 1.0 mL final volume) at room temperature. After 30 min, the absorption at 515 nm was measured in reference to a blank by a Shimadzu UV-1700 spectrophotometer. The results were expressed in terms of the percentage decrease of the initial DPPH^•^ radical absorption by the test samples [14,15,16].

### 3.5. Determination of ABTS^•+^ Scavenging Capacity

The determination of ABTS^•+^ solution scavenging capacity was estimated as previously reported [14,15,16], with slight modifications. The ABTS radical cation was generated by reacting ABTS (2,2′-azinobis-(3-ethylbenzothiazolin-6-sulfonic acid); 7.0 mM) and potassium persulfate (2.45 mM). The mixture was allowed to stand in the dark at room temperature for 12–16 h. Thus, the ABTS^•+^ solution was diluted with PBS (pH 7.4) in order to reach an absorbance of 0.70 at 734 nm. Powders of investigated materials (1.0, 2.0, and 5.0 mg) were directly added to the diluted ABTS^•+^ solution (1.0 mL final volume). After 6 min of incubation, the absorption at 734 nm was measured by a Shimadzu UV-1700 spectrophotometer in reference to a blank. The results were expressed in terms of the percentage decrease of the initial ABTS^•+^ absorption by the test samples.

### 3.6. Cell Culture and Cytotoxicity Assessment

Cytotoxicity was measured via the MTT (3-(4,5-dimethyl-2-thiazolyl)-2,5-diphenyl-2H-tetrazolium bromide) cell viability assay using the murine fibroblast NIH-3T3 cell line, human keratinocyte HaCaT, and neuroblastoma SH-SY5Y cell lines. All the cell lines were grown in Dulbecco’s Modified Eagle Medium supplemented with 10% fetal bovine serum, 50.0 U/mL penicillin, and 100.0 μg/mL streptomycin, at 37 °C in a humidified atmosphere containing 5% CO_2_. Powders of each synthesized material (1.0 and 2.0 mg) were placed in 24-well plates, and the cells were seeded (1.0 × 10^5^ cells/well). After 48 h of incubation, cells were treated with MTT (500 μL; 0.50 mg/mL), previously dissolved in culture media, for 2 h at 37 °C in a 5% CO_2_ humidified atmosphere. The MTT solution was then removed and DMSO was added to dissolve the originated formazan. Finally, the absorbance at 570 nm of each well was determined using a VictorIII Perkin Elmer fluorescence and absorbance reader.

Cell viability was expressed as a percentage of mitochondrial redox activity of the cells directly exposed to powders compared to an unexposed control [15]. Tests were carried out by performing six replicate (*n* = 6) measurements for three samples of each extract (in total: 6 × 3 measurements).

### 3.7. ATR-FTIR Analysis

Treated and untreated cells underwent spectroscopic analysis by ATR-FTIR [33]. Since the degree of hydration influences the spectral characteristics of the main cellular components, cells were in the form of an anhydrous bio-film favoring the recording of the absorption spectra in the 700–4000 cm^−1^ region. The SH-SY5Y cells, seeded at a density of 1.5 × 10^6^ cells/well, were treated with SiO_2_-CGA, 5 wt% and SiO_2_-CGA, 15 wt% (2.0 mg). After 72 h exposure, cells were harvested by centrifugation at 200× *g* and 4 °C and washed (2×) with an aqueous solution of sterile 0.9% NaCl. The pellet was well suspended in NaCl solution (0.9%, 300 μL) and then placed on a glass slide. After 12 h of drying, a cell smear was analyzed by ATR (attenuated total reflectance, AIM-8800, Shimadzu, Tokyo, Japan) by setting the parameters of observation to 65 scans and 8 cm^−1^ resolutions. The spectra were then processed with the software IRsolution (1.30 Version, Shimadzu, Tokyo, Japan).

## 4. Conclusions

The possibility of combining the versatility of sol–gel technology with the important biological properties of natural phenols, well known for their antioxidant efficacy, represents a new challenge. Herein, chlorogenic acid, broadly distributed in the plant kingdom and mainly abundant in coffee beans and in some organs of plants belonging to Asteraceae family, was embedded into a silica matrix providing new bioactive and antioxidant materials. The biocompatibility of the synthetized materials seemed to strongly depend on the amount of entrapped phenol. Cell-specific effects were observed. In particular, when hybrids with the highest dose of chlorogenic acid were tested towards tumorigenic cells, cytotoxicity was more pronounced. Data from ATR analysis suggested the occurrence of apoptosis. These preliminary acquired data lay the foundation to further analysis aimed at deeply investigating the suitable target of use of these new organic-inorganic hybrids, mainly based on the amount of the phenol therein, for a their safe and effective future application.
